# Division-Independent Differentiation of Muscle Stem Cells During a Growth Stimulus

**DOI:** 10.1093/stmcls/sxad091

**Published:** 2023-12-08

**Authors:** Ahmed Ismaeel, Jensen Goh, C Brooks Mobley, Kevin A Murach, Jamie O Brett, Antoine de Morrée, Thomas A Rando, Charlotte A Peterson, Yuan Wen, John J McCarthy

**Affiliations:** Department of Physiology, University of Kentucky, Lexington, KY, USA; Center for Muscle Biology, University of Kentucky, Lexington, KY, USA; Department of Physiology, University of Kentucky, Lexington, KY, USA; Center for Muscle Biology, University of Kentucky, Lexington, KY, USA; School of Kinesiology, Auburn University, Auburn, AL, USA; Center for Muscle Biology, University of Kentucky, Lexington, KY, USA; Department Health, Human Performance, & Recreation, University of Arkansas, Fayetteville, AR, USA; Department of Neurology and Neurological Sciences, Stanford University School of Medicine, Stanford, CA, USA; Department of Neurology and Neurological Sciences, Stanford University School of Medicine, Stanford, CA, USA; Department of Neurology and Neurological Sciences, Stanford University School of Medicine, Stanford, CA, USA; Broad Stem Cell Research Center, University of California Los Angeles, Los Angeles, CA, USA; Center for Muscle Biology, University of Kentucky, Lexington, KY, USA; Department of Physical Therapy, University of Kentucky, Lexington, KY, USA; Department of Physiology, University of Kentucky, Lexington, KY, USA; Center for Muscle Biology, University of Kentucky, Lexington, KY, USA; Department of Physiology, University of Kentucky, Lexington, KY, USA; Center for Muscle Biology, University of Kentucky, Lexington, KY, USA

**Keywords:** adult stem cells, cell division, DNA replication, single-cell gene expression analysis

## Abstract

Adult muscle stem cells (MuSCs) are known to replicate upon activation before differentiating and fusing to regenerate myofibers. It is unclear whether MuSC differentiation is intrinsically linked to cell division, which has implications for stem cell population maintenance. We use single-cell RNA-sequencing to identify transcriptionally diverse subpopulations of MuSCs after 5 days of a growth stimulus in adult muscle. Trajectory inference in combination with a novel mouse model for tracking MuSC-derived myonuclei and in vivo labeling of DNA replication revealed an MuSC population that exhibited division-independent differentiation and fusion. These findings demonstrate that in response to a growth stimulus in the presence of intact myofibers, MuSC division is not obligatory.

## Graphical Abstract

**Graphical Abstract:**
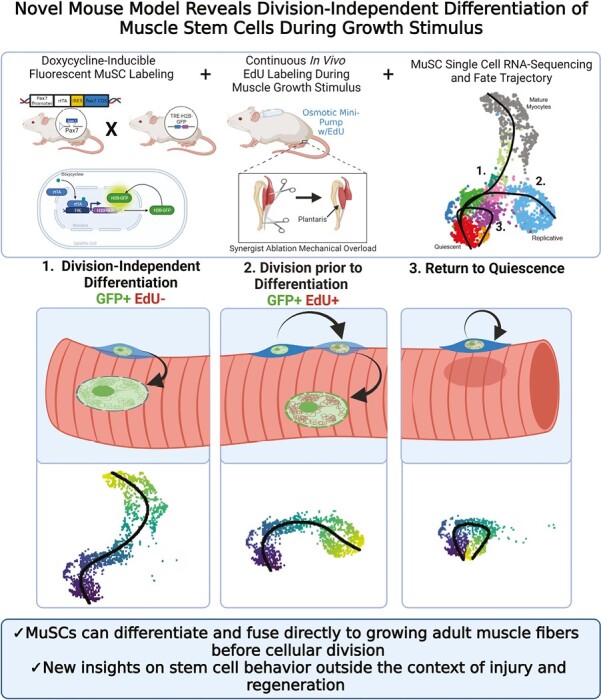

**Significance Statement**

A central dogma in the understanding of stem cell behavior is the intrinsic coupling of cell division and differentiation. Using in silico and in vivo lineage tracing strategies, we identify a subpopulation of adult muscle stem cells that differentiate without undergoing any DNA synthesis in response to a growth stimulus.

## Introduction

The coordination of cell division and differentiation is critical to the proper maintenance of tissue stem cell pools. Adult skeletal muscle stem cells (MuSCs) are typically quiescent but activate and participate in tissue repair after injury.^1-3^ Activated MuSCs first proliferate, then differentiate and fuse to form immature myocytes, but a portion return to quiescence as part of the self-renewal process.^4^ Studies of MuSCs are primarily performed during postnatal development or adult muscle regeneration following massive injury and degeneration.^5-7^ There is growing appreciation that adult uninjured skeletal muscle may exhibit different mechanisms of MuSC regulation, especially during a mechanically induced growth stimulus.^8-10^ Although it is assumed that adult MuSC dynamics follow the same stepwise process observed during development and regeneration, the presence of the intact myofibers provides both membrane-bound and secreted factors that impact the MuSC niche.^1,11-13^

High-resolution transcriptomic methods, such as single-cell RNA-sequencing (scRNA-seq), have proved immensely useful for understanding the heterogeneity and diversity of the different mononuclear cell populations residing in muscle, including MuSCs.^14-17^ To better understand adult MuSC heterogeneity and activation dynamics in response to a growth stimulus in adult muscle, we used scRNA-seq to reveal a previously unappreciated trajectory inference which suggests a subpopulation of adult MuSCs that do not undergo replication, but rather directly differentiate and fuse into existing myofibers. We confirm this cell trajectory using a novel mouse model to lineage trace adult MuSC-derived myonuclei via GFP labeling. We show that during the early response to mechanically induced growth, a significant proportion of GFP+ MuSC-derived myonuclei differentiate and fuse without having undergone DNA replication.

## Materials and Methods

### Experimental Model Details

#### Mice

Animal procedures were conducted in accordance with institutional guidelines approved by the Institutional Animal Care and Use Committee of the University of Kentucky. Adult *Pax7*^*rtTA/+*^ mice (*Pax7*^*rtTa*^) on a C57BL/6 background were generated as previously described.^18^ Briefly, the *Pax7*^*rtTA*^ mouse was generated by inserting a bicistronic cassette containing the reverse tetracycline transactivator-M2 (rtTA-M2) coding sequence with an IRES followed by the *Pax7* coding sequence in frame with the endogenous *Pax7* translational initiation codon of exon 1 (Supplementary Fig. S1A). This targeting strategy permitted the endogenous *Pax7* gene promoter to drive MuSC-specific expression of the *rtTA-M2* while preserving *Pax7* expression. The *Pax7*^*rtTA/+*^ mouse was crossed with the Tg(tetO-HIST1H2BJ/GFP) (TRE-H2B-GFP) transgenic mouse (The Jackson Laboratory, stock No: 005104) to generate the *Pax7*^*rtTA*^; TRE-H2B-GFP mouse. The *Pax7*^*rtTA*^; TRE-H2B-GFP mouse allowed MuSC nuclei to be stably labeled with GFP following doxycycline treatment (Supplementary Fig. S1B). *Pax7*^*rtTA*^; TRE-H2B-GFP offspring were genotyped as previously described,^18^ with mice of the correct genotype housed in a temperature and humidity-controlled room, maintained on a 14:10 h light-dark cycle with food and water provided ad libitum.

#### Effective In Vivo Labeling of MuSC Nuclei Using the *Pax7*^*rtTA*^; TRE-H2B-GFP Mouse

**Figure 1.**
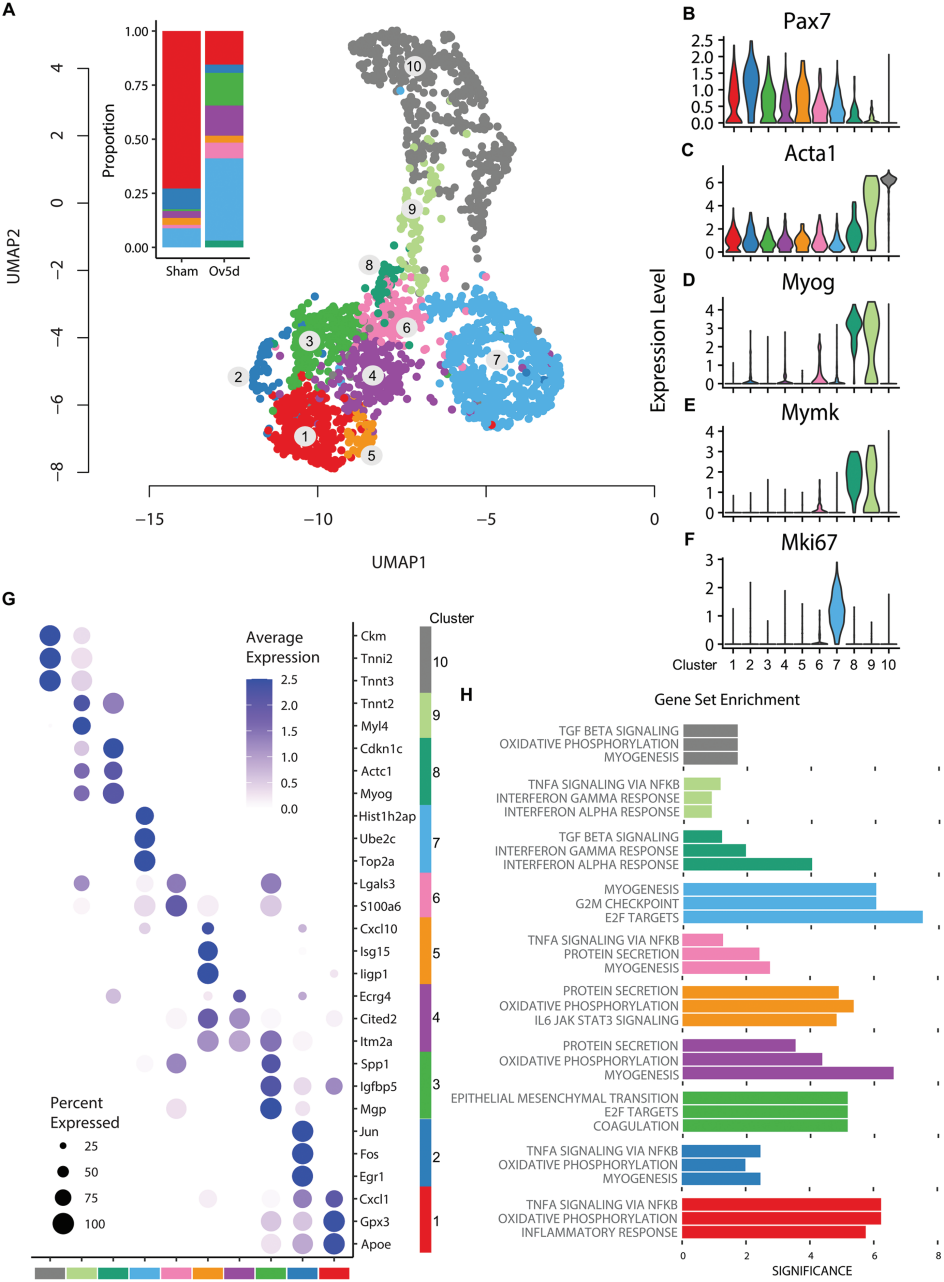
Extensive adult MuSCs heterogeneity in response to a growth stimulus. (**A**) Unbiased clustering and 2D UMAP representation of adult MuSCs in plantaris muscles that were subjected to Sham surgery (*n* = 8) or 5 days of a MOV growth stimulus (Ov5d, *n* = 8). Inset shows proportions of adult MuSC subpopulations (excluding mature myocyte cluster 10). (**B-F**) Expression of key marker genes in each of the cell subpopulations. (**G**) Dot plot of top 3 significant marker genes for each cluster. (**H**) single cell gene set enrichment analysis (GSEA) of significant marker genes for each cell subpopulation. All clusters are color matched.

Fluorescence-activated cell sorting (FACS) was performed on whole-muscle cell suspensions from doxycycline-treated *Pax7*^*rtTA*^; TRE-H2B-GFP mice (*n* = 3), showing 74% of VCAM+ cells were GFP+, with no GFP+ cells detected in vehicle-treated mice (Supplementary Fig. S1C, S1D). When serial muscle sections of doxycycline-treated *Pax7*^*rtTA*^; TRE-H2B-GFP mice were stained for PAX7 or GFP, the abundances of Pax7+ and GFP+ nuclei were comparable (Fig. 1E). Collectively, these results demonstrate the effective labeling of MuSC nuclei with GFP following doxycycline treatment using the *Pax7*^*rtTA*^; TRE-H2B-GFP mouse.

#### Experimental Design

Adult (~6-month old) male and female *Pax7*^*rtTA*^; TRE-H2B-GFP mice were treated with doxycycline hyclate (Sigma, St. Louis, MO) at 2.0 mg/mL in water supplemented with 5% sucrose for 14 days, followed by a 3-day washout period. Following the washout period, mice were randomly assigned to either a sham or synergist ablation surgery group with the plantaris muscle collected after 5 days postsurgery (*n* = 5-8/group).

#### Mechanical Overload Surgery

The synergist ablation surgery places a mechanical overload (MOV) on the plantaris muscle of the lower hind limb that induces a growth response associated with robust MuSC activation, proliferation, and myofiber fusion.^19^ Mice were anesthetized (3% isoflurane with 1.5 L of O_2_ per minute) and placed in sternal recumbence, where a small incision was made on the dorsal aspect of the lower hind limb, with the distal portion of the gastrocnemius muscle tendon and approximately half of the soleus muscle carefully excised. No apparent damage was observed to the neural and/or vascular supply of the plantaris muscle following the surgery. At the designated time point postsurgery, mice were euthanized via CO_2_ inhalation followed by cervical dislocation with muscle collected and prepared accordingly for downstream analyses. The sham surgery consisted of performing the incision and carefully probing the plantaris tendon, without removing the tendon or muscle.

### Method Details

#### Fluorescence-Activated Cell Sorting

FACS was used to assess the effectiveness of the *Pax7*^*rtTA/*^; TRE-H2B-GFP mouse to GFP-label MuSC nuclei following doxycycline administration and for scRNA-seq experiment. To generate a single-cell suspension, fresh plantaris muscle was first digested using collagenase II, followed by collagenase II (Worthington-Biochem, Lakewood, NJ) and dispase (Sigma-Aldrich) treatment. The cell suspension was serially passed through a 70- and 40-µm filter and incubated with antibodies against VCAM, CD31, CD45, and Sca1 (Biolegend, San Diego, CA) at 4 °C. A biotinylated secondary antibody was used for VCAM, while all other antibodies were PE-Cy7-conjugated. Immediately following the incubation, cells were pelleted (500 × *g* for 5 minutes), re-suspended in FACS sorting buffer, and sorted using an iCyt FACS machine (Sony Biotechnology, Champaign, IL).

#### Single-Cell RNA-Sequencing

Plantaris muscle, single-cell suspensions were prepared as described above from doxycycline-treated *Pax7*^*rtTA*^; TRE-H2B-GFP mice subjected to either sham surgery (*n* = 8) or 5 days of a growth stimulus (*n* = 8). VCAM+, CD31−, CD45−, and SCA1− cells were directly sorted into reverse transcription buffer after being cleared of debris, dead cells (DAPI+), and doublets via FACS. Cells were then loaded into the 10X Chromium Controller using the Single Cell 3’ reagent kit according to the manufacturer’s protocol. RNA sequencing libraries were prepared and sequenced on the Illumina NextSeq 500 platform, achieving a minimum of 40 000 reads per sample.

#### Bioinformatics Analysis of scRNA-seq Data

Read sequences in fastq format were aligned to the mouse genome (mm10 build 2020-A, Ensembl release 98) using Cell Ranger v6.0.0 (10X Genomics). The filtered count matrices from Cell Ranger, including gene symbols and cell barcodes, were imported into R v4.0.2 programming environment using Seurat v4.0.2^20^ and normalized using SCTransform.^21^ Both datasets were integrated using the anchor-based strategy implemented in Seurat.^22^ Dead or dying cells with 10% or greater mitochondrial-encoded transcripts were removed from further analysis. Doublet cells were identified using DoubletFinder v2.0.3^23^ for the integrated dataset and removed from further analysis. Unbiased cell clustering was performed on the integrated dataset using the first 50 principal components at a resolution of one. Subsequent analyses were restricted to the subset of myogenic cells (positive for *Pax7* or adult skeletal muscle myosin heavy chain and negative for *Pdgfra*). The myogenic cells were extracted as a subset from the integrated dataset and unbiased clustering was performed at a resolution of 2 using the first 50 principal components. RNA counts were normalized and scaled before gene expression was analyzed using FindAllMarkers function with the following parameters: “only.pos = TRUE, min.pct = 0.5, logfc.threshold = 0.5.” Single-cell gene set enrichment analysis (scGSEA) was performed using Singleseqgset v.1.2.9 and the mouse Hallmark gene set from msigdbr v7.5.1.^24^ Significance level for both marker genes and scGSEA was set at an adjusted *P* value <.05. The barcodes representing the subset of myogenic cells along with their uniform manifold approximation and projection (UMAP) and principle component analysis (PCA) embeddings were converted to SingleCellExperiment v1.18.0^25^ format using the SeuratWrappers v0.3.0 package and imported into SlingShot v2.4.0^26^ for pseudotime calculations and lineage analysis. The SlingShot method was chosen due to its high performance, robustness, and usability.^27^ Lineage-specific gene expression was analyzed using the tradeSeq v1.10.0^28^ package by fitting a negative binomial generalized additive model (fitGAM function). The optimal number of basis functions, or knots, was empirically determined to be 12 using the evaluateK function, which minimized the relative Akaike Information Criterion (AIC) score in the range from 6 to 15 knots. Evaluation was performed in 4 replicates using seed values from 5 to 8 to assess the stability of the relative AIC. Early lineage-associated genes were analyzed using the startVsEndTest, which tests the significance of the Wald statistic for log fold difference of a gene at the end pseudotime (4.5) compared to the start pseudotime (2.5). A pseudotime of 4.5-5 represented the midpoint of all 3 lineages. Pairwise differential analysis contrasting genes associated with each lineage was performed using the diffEndTest. Pathways analysis of significant lineage-specific genes were analyzed and visualized using enrichR.^29-31^

#### Labeling of MuSC Proliferation

EdU (5-ethynyl-2ʹ-deoxyuridine) incorporation was used to detect MuSC proliferation in response to a growth stimulus. EdU (Carbosynth, Berkshire, UK) was prepared in 1X phosphate-buffered saline (PBS) solution and administered via intraperitoneal (IP) injection at a dose of 0.1 mg per 30 g of body weight as previously described^32^ immediately prior to subcutaneous implantation of mini osmotic pump (Azlet mini model 1007D), which releases EdU continuously for up to 7 days (Azlet Osmotic Pumps, Cupertino, CA). To determine the saturating EdU dose, mice were subjected to 7 days of MOV by synergist ablation, along with implantation of mini osmotic pumps administering 0, 6.25, 12.5, 25, or 50 mg/kg/day EdU. For the 5-day MOV experiments, 25 mg/kg/day EdU was delivered by mini osmotic pumps.

#### Immunohistochemistry

Immunohistochemistry (IHC) analysis was performed on the plantaris muscle to determine the effectiveness of the *Pax7*^*rtTA*^; TRE-H2B-GFP mouse to label MuSC nuclei with GFP, track MuSC fusion to myofibers and the level of MuSC replication based on EdU incorporation. Immediately following euthanasia, excised plantaris muscles were immediately weighed, covered in Tissue-Tek optimal cutting temperature compound (Sakura Finetek, Torrance, CA), pinned at resting length to aluminum-covered cork block, frozen in liquid nitrogen-cooled isopentane and then stored at −80 °C until tissue sectioning. At the time of sectioning, muscle samples were trimmed to mid-belly, oriented upright with sections cut at −20 °C at a thickness of 7 µm. Tissue sections were air-dried >1 hour at room temperature, fixed with 4% paraformaldehyde (VWR, Radnor, PA), permeabilized using 0.1% Triton X-100 (Sigma-Aldrich), and blocked for endogenous peroxidases using 3% H_2_O_2_ (Sigma-Aldrich). Sections were then blocked in a cocktail of 1% bovine serum albumin (Thermo Fisher Scientific, Waltham, MA), and 3% mouse-on-mouse blocking reagent (VectorLabs, Burlinghame, CA). Following these blocking steps, sections were incubated in a 1:100 dilution of either an anti-rabbit (Abcam, Cambridge, UK) or anti-mouse (Developmental Studies Hybridoma Bank, Iowa City, IA) primary antibody against dystrophin overnight at 4 °C, followed by the appropriate secondary antibodies (1:200) and 1:10 000 dilution of 4ʹ,6‐diamidino‐2‐phenylindole (DAPI; Thermo Fisher Scientific). EdU incorporation was detected as previously described,^32^ which involved copper‐mediated Click‐iT chemistry followed by a TAMRA azide secondary antibody (Thermo Fisher Scientific). Since the EdU detection cocktail quenched the GFP signal, GFP was rescued in EdU‐labeled muscle sections using a 1:200 dilution of biotinylated GFP antibody in 2% bovine serum albumin (Rockland Immunochemicals, Pottstown, PA) along with a 1:500 dilution of goat anti‐mouse streptavidin horseradish peroxidase secondary (Thermo Fisher Scientific), followed by tyramide signal amplification (Thermo Fisher Scientific) for *Pax7*^*rtTA*^; TRE-H2B-GFP mice. Sections were mounted using a 50/50 solution of PBS and glycerol. For PAX7 staining, epitope retrieval was performed with sodium citrate buffer (pH 6.8) at 92 °C, and sections were incubated in a 1:100 dilution of anti-mouse PAX7 (Developmental Studies Hybridoma Bank), followed by anti-mouse streptavidin horseradish peroxidase secondary and tyramide signal amplification (Thermo Fisher Scientific).

#### Image Acquisition and Quantification

For IHC, images were captured at ×20 magnification using either a Zeiss upright fluorescent microscope (Zeiss AxioImager M1 Oberkochen, Germany) or a Olympus BX61VS Upright Fluorescent Microscope (Evident Scientific, Bethlehem, PA). Whole plantaris muscle sections were obtained using the mosaic function in Zeiss Zen 2.3 or Olympus whole-slide scan and converted to Open Microscopy Environment compatible TIFF format.^33-35^ Only GFP+ myonuclei within dystrophin border (either EdU+ or EdU−) were counted.

#### Statistics

Cluster-specific gene expression and GSEA were using the Wilcoxon Rank Sum test comparing each cluster with cells in all other clusters. Lineage-specific gene expression analysis was determined using the Wald test after fitting a negative binomial generalized additive model to smooth the scRNA-seq data. Pathways enrichment analysis using enrichR used Fisher’s exact test. All *P* values were adjusted for false discovery using the Benjamini-Hochberg method and significance was set at adjusted *P* < .05 for all analyses. The difference in the number of GFP + EdU− and GFP + EdU+ nuclei was assessed using a paired *t* test, and significance was set at *P* < .05.

## Results

### scRNA-seq Reveals Extensive MuSC Heterogeneity in Response to a Growth Stimulus

To profile adult MuSCs during a growth stimulus, we performed scRNA-seq on VCAM+ MuSCs isolated from plantaris muscles of mice subjected to either sham surgery or 5 days of MOV. The 5-day time point was chosen to maximize the abundance of activated MuSCs prior to the dramatic fusion to myofibers that occurs by 7 days of MOV.^36,37^ The total number of MuSCs analyzed by scRNA-seq in sham and MOV conditions were 604 and 1746 cells, respectively. UMAP was used to construct a visual representation of gene expression topology (Fig. 1A). Unsupervised clustering grouped cells into 10 subpopulations based on their transcriptional programs (Supplementary File 1), including a cluster resembling mature myocytes as indicated by adult myosin heavy chain (MyHC) expression (cluster 10) and lack of *Pax7* expression (Fig. 1B). The presence of a mature myocyte cluster despite size-filtering during cell isolation is consistent with previous reports.^38,39^ We excluded clusters 9 and 10 which expressed high levels of terminal differentiation genes (*Acta1*, Fig. 1C) to calculate the relative abundance of MuSCs. Under conditions without a growth stimulus (Sham), clusters 1 and 2 contributed over 80% of the MuSC pool, with other clusters comprising only minor proportions (Fig. 1A, inset). Cells in clusters 1 and 2 displayed a profile consistent with MuSC quiescence (high *Pax7*). Cells in cluster 1 also expressed high levels of *Chrdl2*, a marker of MuSC quiescence^40^ (Supplementary Fig. S2A). After 5 days of MOV, however, the proportion of MuSCs in the remaining clusters increased, resulting in the emergence of a more heterogenous population of MuSCs. In addition to high *Pax7* expression, clusters 1, 2, and 3 were marked by high expression of other genes associated with MuSC quiescence, including *Apoe* and *Notch,* as well as *Col5*.^41-43^ MuSCs in cluster 2 also expressed immediate-early response genes (*Fos, Jun,* and *Egr1*),^44,45^ while MuSCs in cluster 3 expressed other markers of activating MuSCs (*Islr* and *Mgp*).^46,47^ The subpopulation of MuSCs in cluster 4 expressed genes associated with MuSC self-renewal (*Sox4*).^48^ Additionally, MuSCs in clusters 4 and 5 expressed genes associated with polarity and asymmetric division (*Dag1*)^49^ and tetraspanin genes (*Cd63* and *Cd9*). MuSCs in cluster 6 expressed high levels of genes encoding ribosomal proteins (*Rps2* and *Rpl3*) and genes expressed during the pre-fusion phase (*Myof*).^50^ Furthermore, a subset of MuSCs in clusters 8 and 9 displayed high levels of myogenic genes (*Myog*, Fig. 1D; *Itga7*, Supplementary Fig. S2B; and *Myod1*, Supplementary Fig. S2C) and the fusogenic genes *Mymx and Mymk* (Fig. 1E), which are both required and sufficient for myogenic fusion.^51-54^ Expression levels of the myogenic regulatory factor *Myf5* were low in clusters 8 and 9 (Supplementary Fig. S2D). Finally, in response to the growth stimulus, cluster 7 contributed the greatest proportion (~40%) to the MuSC pool. MuSCs found in cluster 7 expressed genes characteristic of proliferating cells, including high expression of *Mki67* (Fig. 1F), cell cycle genes (*Cdk1* and *Ccna2*), and DNA metabolism genes expressed at the beginning of S phase (*Rad51ap1*, *Rfc4*, and *Rrm2*).^55^

### scGSEA Identifies Heterogenous Transcriptional Programs of MuSC Clusters During a Growth Stimulus

To better understand the functional profile of each of the subpopulations of MuSCs following 5 days of MOV, we performed scGSEA on the significant genes for each cluster. Clusters 1 and 2 were characterized by upregulation of genes associated with oxidative phosphorylation, consistent with dependence of quiescent MuSCs on mitochondrial fatty acid oxidation and oxidative phosphorylation.^56^ Clusters 1 and 2 were also marked by upregulation of TNF-α signaling via NF-κB and inflammatory response, with top differentially expressed genes (DEGs) in cluster 1 *Cxcl1* and *Gpx3,* (Fig. 1G, 1H) consistent with the quiescent MuSC molecular signatures of inflammation and antioxidants.^42^ A subpopulation of MuSCs in cluster 3 was characterized by upregulated genes associated with epithelial-mesenchymal transition, E2F targets, and coagulation. The top DEGs in this cluster, *Spp1* and *Mgp*, are known to influence the myogenic process by regulation of extracellular matrix genes.^47,57^ Clusters 4, 5, and 6 were characterized by upregulation of protein secretion (secretion of cytokines), and clusters 5 and 6 were also enriched for signal transduction pathways. The top DEGs in clusters 4 and 6 included membrane-related genes (*Itm2a* and *Lgals3*), and DEGs in cluster 5 included *IL6/JAK/STAT3* signaling genes such as interferon-stimulated gene 15 (*Isg15)* and interferon-inducible GTPase 1 (*Iigp1*). Upregulated genes in the cluster of MuSCs indicative of active replication (cluster 7) were associated with G2/M DNA damage and cell cycle checkpoint, a defining feature of replicating cells. The top DEGs (*Hist1h2ap, Ube2c,* and *Top2a)* are related to cell cycle, cell proliferation, and DNA metabolism.^58^ MuSCs in cluster 8 demonstrated upregulated genes associated with TGF-β signaling and interferon-γ/α response. Top DEGs in this cluster included the lineage commitment myogenic regulatory factor *Myog,* as well as the negative regulator of cellular proliferation, *Cdkn1c*.^59,60^ Finally, MuSCs in cluster 9 also expressed genes associated with interferon-γ/α response pathways and TNF-α signaling via NF-κB. This subpopulation of MuSCs also expressed high levels of genes representative of mature myocytes (*Tnnt2* and *Myl4*).

### Cell Fate Decision-Making Occurs in Parallel Rather Than in a Linear Fashion

**Figure 2.**
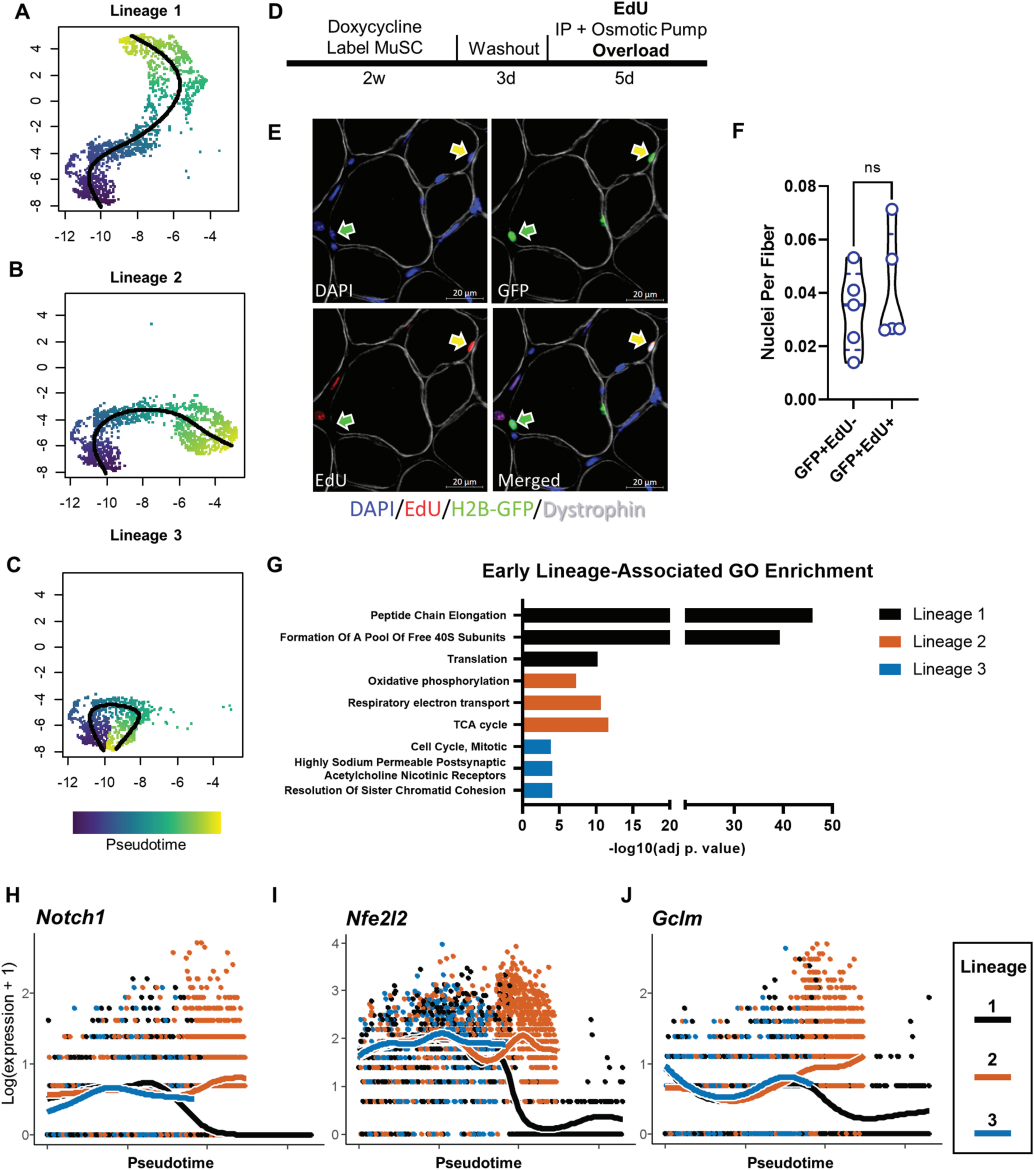
MuSCs directly fuse into muscle cells without undergoing replication depending on cellular metabolism. Trajectory inference delineates 3 distinct lineages of MuSCs progression from quiescence. (**A**) Lineage 1 represents direct commitment and fusion of MuSCs into myocytes. (**B**) Lineage 2 represents replicating MuSCs that replenish tissue MuSC pool. (**C**) Lineage 3 represents MuSCs that return to quiescence or a quiescence-like state. Color bar represents pseudotime of lineage progression. (**D**) Experimental design and labeling strategy to lineage trace MuSCs and determine replication status. (**E**) Representative images of muscle histological cross-sections showing MuSC-derived (green) myonuclei under the sarcolemma (white dystrophin border) with and without EdU incorporation (red). (**F**) Quantification of MuSC-derived myonuclei that either replicated (EdU + GFP+, yellow arrow) or did not replicate previously (EdU-GFP+, green arrow). Difference assessed using a paired *t* test, ns = not significant (*P *> .05). (**G**) Gene ontology (GO) enrichment analysis comparing early lineage-associated genes (pseudotime 2.5-4.5). Gene expression is plotted for cells along each lineage for *Notch1* (**H**), *Nfe2l2* (**I**), and *Gclm* (**J**).

Based on previously reported scRNA-seq analysis of MuSCs during regeneration,^15,38,39^ we anticipated the progression of MuSCs from quiescence to replication, then differentiation, and finally fusion into mature myocytes. However, the organization of the subpopulations of MuSCs did not confirm this expectation. Instead, trajectory analysis with SlingShot^26^ identified 3 distinct lineage inferences (Fig. 2A-2C). Lineage 1 represents a trajectory inference of quiescent MuSCs leading directly to commitment and fusion into the mature myocyte cluster (Fig. 2A). Lineage 2 represents a trajectory inference path leading to replication, marked with high expression of *Mki67*, a commonly used marker of DNA synthesis and cell division (Fig. 2B). Finally, lineage 3 represents a trajectory inference path that leads from activation back to a quiescence-like state (Fig. 2C). Taken together, in response to a mechanical growth stimulus, adult MuSCs appear to make one of 3 independent cell fate decisions. This finding is in stark contrast to the well-established sequential response of MuSC entry into G1, progression through S, G2, and M phases prior to making the decision to exit the cell cycle and returning to G0. Notably, this trifurcation revealed by our trajectory analysis is also different from the bifurcating trajectory that has been previously described in muscle regeneration.^38,61^ These data suggest that adult MuSCs are capable of directly differentiating without replicating during MOV.

### A Subset of MuSCs Directly Differentiate and Fuse Without Proliferation

To verify the presence of lineage inference 1 MuSCs, we tracked adult MuSC division and fusion into the myofiber following a growth stimulus. We fluorescently labeled adult MuSC nuclei using a recombinant histone-EGFP fusion protein by crossing a MuSC-specific Tet-ON mouse^18^ (*Pax7*^*rtTA*^, Supplementary Fig. S1A) to a tetracycline-responsive nuclear-localized GFP reporter mouse Tg(tetO-HIST1H2BJ/GFP) (TRE-H2B-GFP) to generate the *Pax7*^*rtTA*^; TRE-H2B-GFP mouse (Supplementary Fig. S1B). Following dox administration via drinking water (2 mg/mL), >75% MuSCs were GFP+ as assessed by FACS (Supplementary Fig. S1C, S1D) and PAX7 IHC (Supplementary Fig. S1E). In contrast to models using the Cre-loxP system, an advantage of the Tet-ON system is non-constitutive expression, as induction of H2B-GFP only occurs during dox administration. To label replicating adult MuSCs, dox-treated *Pax7*^*rtTA*^; TRE-H2B-GFP mice were administered a loading dose of EdU by IP injection, prior to completion of the MOV surgery, and then continuously provided EdU via subcutaneous mini osmotic pump (25 mg/kg/d) for 5 days (Fig. 2D). The loading dose ensures that EdU is bioavailable before the growth stimulus is applied to the muscle. The continuous delivery at a saturating dose through the mini osmotic pump guarantees no replication event is missed through the duration of the growth response. This administration method was required to capture all dividing adult MuSCs because EdU is cleared from the bloodstream within a few hours after IP injection, and daily IP delivery leads to gaps in EdU bioavailability and incomplete labeling.^62,63^ We determined that 25 mg/kg/day achieved saturating levels of the thymidine analog (Supplementary Fig. S1F), with ~90% of Pax7+ nuclei labeled during 7 days of MOV (Supplementary Fig. S1G). Using this lineage tracing and DNA synthesis labeling strategy, we could distinguish between adult MuSCs that had undergone at least one round of cell division and those that had not following 5 days of MOV. Combined with labeling of the sarcolemma using anti-dystrophin immunofluorescence, we quantified adult MuSCs that fused into the myofiber with (GFP+/EdU+) or without (GFP+/EdU−) previous cell division (Fig. 2E). We found a similar number of newly derived (GFP+) myonuclei that had never undergone cell division (EdU−) as those that had (EdU+) following a 5-day growth stimulus (Fig. 2F). The results from in vivo lineage and cell division tracing were consistent with the scRNA-seq trajectory analysis, confirming that a population of GFP+ adult MuSCs undergo division-independent differentiation and fusion into the myofiber.

### Pathway Analysis Reveals Early Lineage-Associated Transcriptional Programs

To gain insight into potential lineage-associated gene programs, we performed gene expression and lineage inference correlation analysis during early pseudotime using tradeSeq.^28^ We chose a pseudotime range between 2.5 and 4.5, which represents the latter portion of the first half of the trajectory inference, during which the trifurcation is predicted. Figure 2G shows the top enriched pathways for the early determinant genes for each of the 3 lineage inferences. Pathways related to translation were most enriched for lineage inference 1 (direct fusion), while mitochondrial and aerobic respiratory chain and ATP synthesis pathways were enriched for lineage inference 2 (replicating). Early determinant genes for lineage inference 3 (return to quiescence) were enriched for cell cycle pathways. To characterize the transcriptomic signature that distinguishes adult MuSCs that directly fuse from those that divide, we performed differential expression analysis contrasting lineage inferences 1 and 2 across pseudotime. Interestingly, genes downregulated in lineage inference 1 compared to lineage inference 2 included *Notch1* (Fig. 2H), a known regulator of MuSC fate determination,^64^ as well as the antioxidant/glutathione metabolism genes *Nfe2l2* (Fig. 2I), also known as *Nrf2,* and *Gclm* (Fig. 2J), which have recently been reported to affect MuSC proliferation and function.^65^

## Discussion

In this study, we demonstrate that adult MuSCs are capable of division-independent differentiation in response to a growth stimulus both by computational inference and direct in vivo tracing of cell division and lineage. Traditionally, stem cell differentiation has been thought to start with commitment, followed by proliferation, and finally terminal differentiation.^66^ However, recent studies of hematopoietic stem cells (HSCs) have challenged the traditional stem cell activation model and provided evidence that HSCs exhibit division-independent differentiation.^67-69^ Similarly, *Drosophila* follicle stem cells and mammalian epidermal and intestinal stem cells can differentiate without division, which was predicted to result in proliferative competition among these stem cells.^70^ In muscle, the presence of the intact differentiated myofibers in close contact with MuSCs likely facilitates direct heterotypic fusion. While data from previous reports have hinted at the presence of a direct differentiation trajectory,^71-72a^ further investigation has been limited by a lack of tools to assess MuSC population dynamics. In this report, our novel mouse model for inducible, temporally controlled MuSC labeling has allowed us to perform, for the first time, lineage tracing studies with independence from thymidine analog incorporation. There are 3 features of the *Pax7*^*rtTA*^; TRE-H2B-GFP mouse that ensured the GFP+ myonuclei were derived from newly fused MuSCs: 1) induction of the H2B-GFP reporter gene only occurs during doxycycline (dox) administration; dox was withdrawn 3 days prior to MOV; 2) the H2B-GFP fusion protein is stably incorporated into the chromatin; and 3) expression of rtTA is regulated by the *Pax7* promoter which is downregulated prior to myofiber fusion.

MuSCs have long been studied as a model system of the temporal coupling between proliferation and differentiation.^73^ However, MuSC dynamics likely differ depending on the physiological conditions. It is important to note that previous differentiation trajectory analyses that identified a stepwise process of MuSC activation, proliferation, differentiation, and fusion involved models that used toxins to induce extensive muscle injury, such as barium chloride or notexin injection.^38,39,74^ Under these extreme conditions, immense muscle degeneration leads to rapid expansion of the MuSC pool, ensuring sufficient biomass for effective muscle regeneration. In the presence of such extensive necrosis, MuSCs are activated quickly and undergo multiple rounds of proliferation, characteristic of symmetric division prior to differentiation.^75-77^ In regenerating muscle, MuSC division is oriented along “ghost fiber” remnants of injured myofibers and influenced by the regenerating MuSC niche.^8,9^ Proliferating MuSCs initially fuse with each other to form myotubes. A subsequent second wave of fusion to immature myofibers may be related to the peripheral location of newly added myonuclei.^61^ On the other hand, in the absence of fiber degeneration in response to a growth stimulus, adult MuSCs can proliferate between the basal lamina and myofiber sarcolemma, and fuse with the pre-existing myofibers.^8-10^ In the presence of intact myofibers, direct MuSC fusion during growth represents heterotypic fusion (MuSC to myofiber), in contrast to the homotypic fusion (MuSC to MuSC) that characterizes the early phase of regeneration.

Although ~50% of GFP+ MuSC-derived myonuclei differentiated and fused without first dividing, the functional significance of a subset of directly differentiating MuSCs remains unknown. Using our mouse model for dox-inducible fluorescent MuSC labeling along with continuous EdU labeling to assess replication, this study is, to our knowledge, the first observation of division-independent differentiation in muscle in response to a physiological stimulus while maintaining the stem cell pool. Our lineage inference-specific analysis identified that *Notch1* expression is significantly downregulated in the lineage inference leading to direct fusion. Ablation of NOTCH signaling through genetic manipulation induced MuSC to fuse without proliferation, thus causing a depletion of the MuSC pool.^41,71,78,79^ We also found that early genes associated with the lineage inference leading to cell division were significantly enriched for mitochondrial electron transport proteins. Inhibition of Complex IV activity via *Cox10* knockout increased ROS production and led to the direct fusion of MuSCs with the existing myofibers and a depletion of the MuSC pool.^80^ We further found significantly lower expression of antioxidant genes *Nrf2* and *Gclm* in MuSCs with a direct fusion trajectory. The loss of these genes was recently reported to cause defects in S-phase entry in MuSCs.^65^ Notably, a Notch-Nrf2 axis has also been described, as stimulation of NOTCH signaling induces expression of Nrf2 and its target genes.^81^ Furthermore, a functional antioxidant response element in the gene-regulatory region of *Notch1* has been identified, and Nrf2 can also enhance the expression of Notch.^82^ Crosstalk between the Nrf2 and NOTCH pathways^83^ may play a role in the determination of MuSC fate.

The above examples of division-independent adult MuSC differentiation share a common feature: lower proliferative capacity. Thus, one intriguing possibility is that the mechanical loading-induced growth stimulus confers a selective pressure, resulting in some MuSCs undergoing immediate differentiation and fusion into the myofiber, while others proliferate to maintain the MuSC pool. Aside from intrinsic properties of MuSCs, dynamics that dictate division-dependent or independent differentiation may be the result of extrinsic MuSC-niche interactions or exposure to local mitogens, including growth factors or inflammatory cytokines.^84,85^

Our study has limitations. First, selection biases can be introduced into scRNA-seq by FACS sorting. Only selecting for VCAM+ cells precluded the analysis of any MuSC that may have lost VCAM expression if there were such a population. Second, while a continuum of stress states likely exists in which cells can move in either direction over time, a limitation of trajectory inference methods is that the pseudotime simply describes the transition from one end of the continuum to the other. Thus, cell fate progression may appear discrete, and intermediate states can sometimes be seen as final stages.^86^ However, cells may not be restricted to either direction along the projected path from a computational perspective, and directionality is an interpretation based on biological understanding of MuSCs. Still, our in vivo lineage tracing strategies confirmed the computational trajectory inference. Another limitation of the study is our focus on only MuSCs prevented investigation of the role of neighboring cells and extrinsic signals that may affect the MuSC niche and impact MuSC activation, proliferation, and differentiation. Finally, we did not experimentally confirm the contribution of specific factors promoting the direct differentiation of the subset of adult MuSCs in response to a growth stimulus; future studies can build upon this work to identify these factors and the functional significance of MuSC direct differentiation in response to growth stimuli.

## Summary

A skeletal muscle growth stimulus induces heterogeneous subpopulations of MuSCs. Lineage analysis reveals trifurcated cell fate decision-making. An equal proportion of MuSCs differentiate and fuse into the myofibers with and without prior replication as assessed by GFP+ EdU+/− myonuclei.

## Supplementary Material

sxad091_suppl_Supplementary_Material

## Data Availability

Further information and requests for resources and reagents should be directed to and will be fulfilled by the Lead Contact. All scRNA-seq datasets are uploaded to the Gene Expression Omnibus (GEO) under the accession number GSE235102.
