# Correction to: Division-Independent Differentiation of Muscle Stem Cells During a Growth Stimulus

**DOI:** 10.1093/stmcls/sxae021

**Published:** 2024-03-14

This is a correction to: Ahmed Ismaeel, Jensen Goh, C Brooks Mobley, Kevin A Murach, Jamie O Brett, Antoine de Morrée, Thomas A Rando, Charlotte A Peterson, Yuan Wen, John J McCarthy, Division-Independent Differentiation of Muscle Stem Cells During a Growth Stimulus, Stem Cells, 2023;, sxad091, https://doi.org/10.1093/stmcls/sxad091

The originally published version of this article has been corrected.

The following reference and its in-text citation has been added to the article as Reference 72a, as the study in question first proposed the idea that muscle stem cells are capable of myofiber fusion without cell division:

 “Pawlikowski, B., Pulliam, C., Betta, N.D. *et al.* Pervasive satellite cell contribution to uninjured adult muscle fibers. *Skeletal Muscle***5**, 42 (2015). https://doi.org/10.1186/s13395-015-0067-1”.

