# Supplementary material for: Division-Independent Differentiation of Muscle Stem Cells During a Growth Stimulus
Source: Stem Cells. 2023 Dec 8;42(3):266–77. doi: 10.1093/stmcls/sxad091 (PMC10938546; doi:10.1093/stmcls/sxad091)
Supplement: sxad091_suppl_Supplementary_Material [file sxad091_suppl_supplementary_material.zip › sxad091/New Microsoft Word Document.docx]

**Supplemental Figure 1**

**A)** Schematic diagram showing the genetic knock-in of reverse tetracycline trans-activator (*rtTA*) into the mouse *Pax7* locus (*Pax7^rtTA^*). **B)** Breeding of the MuSC-specific rtTA mouse with a nuclear reporter mouse (H2B-GFP) that is under the control of the tetracycline response element (TRE). In the presence of doxycycline, MuSC nuclei are labeled with GFP. **C**) FACS isolation of VCAM+ cells from skeletal muscle after doxycycline treatment demonstrates a large population of GFP+ cells, indicating robust labeling. **D**) Quantification of GFP+ cells indicate roughly 75% of VCAM+ cells are labeled with GFP. **E**) Quantification of MuSCs using PAX7 IHC and GFP fluorescence expressed as nuclei per fiber. **F, G)** EdU+ nuclei counts per fiber (F) and percentage of PAX7+ nuclei that are EdU+ (G) at increasing doses of EdU administered via an osmotic pump during 7 days of MOV.

**Supplemental Figure 2.** Expression of quiescence and myogenic marker genes in each of the cell subpopulations. Expression levels shown for **A)** *Chrdl2*, **B)** *Itga7*, **C)** *Myod1*, and **D)** *Myf5*

**Supplemental Spreadsheets File 1, related to Figures 1 and 2**. Sheet 1: Marker genes for each cell cluster in Sham or 5 days of a growth stimulus induced by MOV of plantaris muscles. Sheet 2: Cell count for each adult MuSC subpopulation. Sheet 3: scGSEA for all subpopulations of MuSCs. Sheet 4: Differentially expressed genes significantly associated with each of the three lineages determined by trajectory inference. Sheet 5: Decision making genes associated with each lineage as determined by association with early (2.5 - 4.5) pseudotime. Sheet 6: Differential gene expression analysis comparing pairwise lineages.
