# Supplementary figures and images for: Division-Independent Differentiation of Muscle Stem Cells During a Growth Stimulus
Source: Stem Cells. 2023 Dec 8;42(3):266–77. doi: 10.1093/stmcls/sxad091 (PMC10938546; doi:10.1093/stmcls/sxad091)

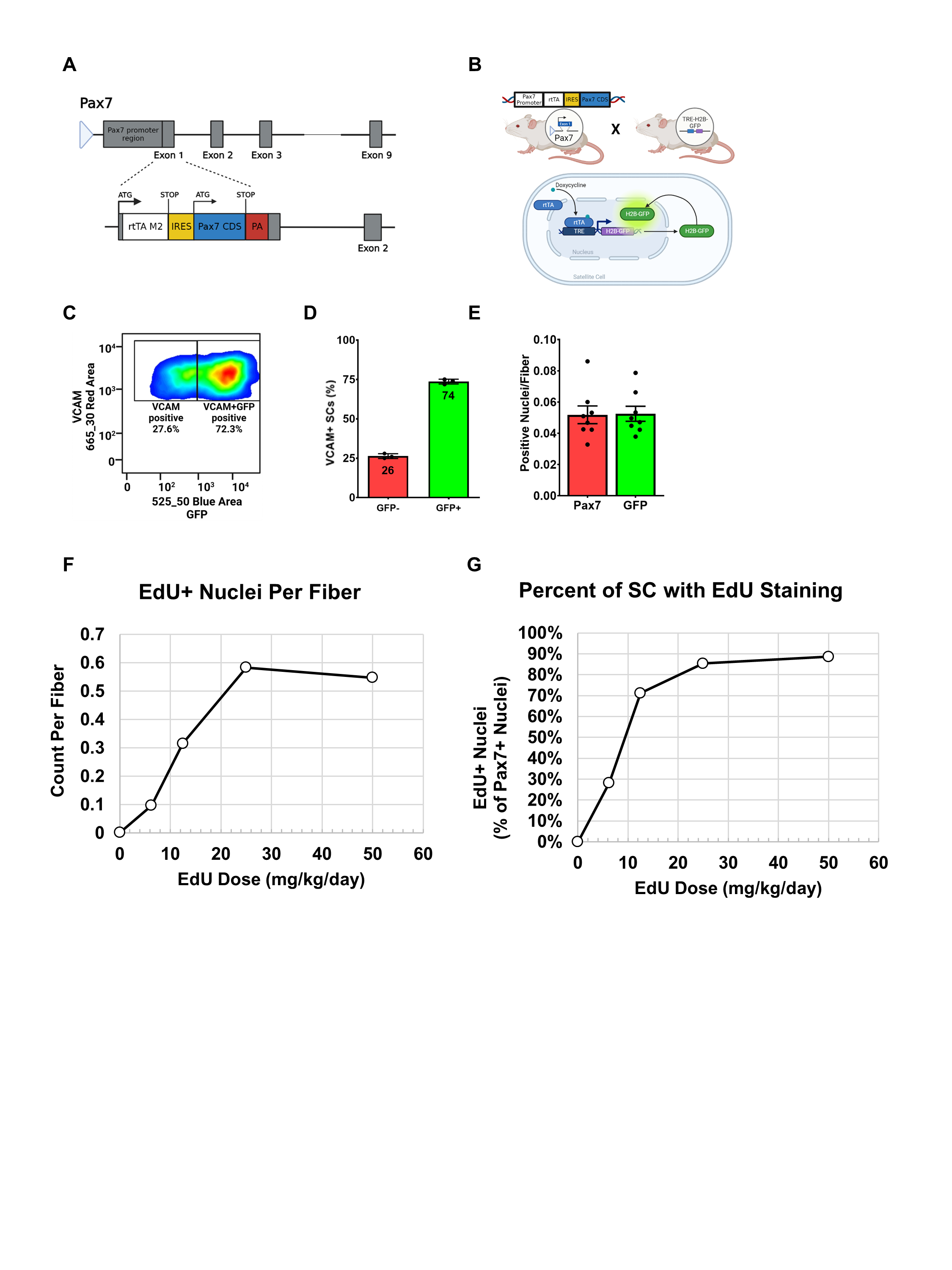

Supplement: sxad091_suppl_Supplementary_Material [file sxad091_suppl_supplementary_material.zip › sxad091/Supplementary Figure 1.tif]

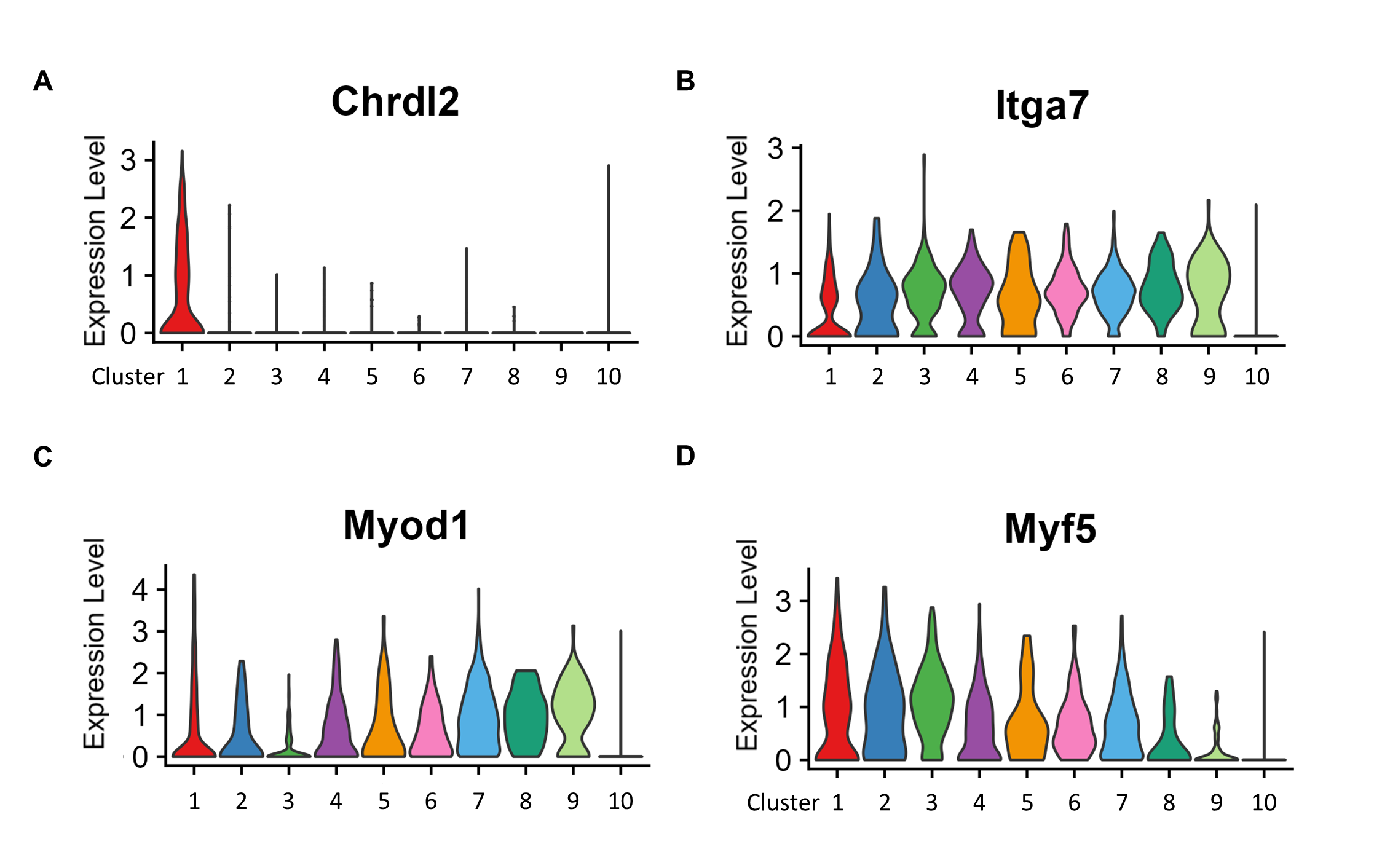

Supplement: sxad091_suppl_Supplementary_Material [file sxad091_suppl_supplementary_material.zip › sxad091/Supplementary Figure 2_R1.tif]
